# Increased Expression of the Δ133p53β Isoform Enhances Brain Metastasis

**DOI:** 10.3390/ijms24021267

**Published:** 2023-01-09

**Authors:** Alexandra N. Boix De Jesus, Ahmad Taha, David Wang, Paulomi M. Mehta, Sunali Mehta, Ashley Reily-Bell, Sasini Polwatta Lekamlage, Adriana Machado Saraiva, Tahmeed Tahmeedzaman, Fouzia Ziad, Ziad Thotathil, Peter Y. C. Gan, Janice Royds, Antony Braithwaite, Noelyn Hung, Tania L. Slatter

**Affiliations:** 1 Department of Pathology, Dunedin School of Medicine, University of Otago, Dunedin 9016, New Zealand; 2 Department of Neurosurgery, Southern District Health Board, Dunedin 9016, New Zealand; 3 Department of Medicine, Dunedin School of Medicine, University of Otago, Dunedin 9016, New Zealand; 4 The Westmead Institute for Medical Research, Sydney, NSW 2145, Australia; 5 Maurice Wilkins Centre for Biodiscovery, University of Otago, Dunedin 9016, New Zealand; 6 Department of Pathology, Waikato District Health Board, Hamilton 3204, New Zealand; 7 Department of Radiation Oncology, Waikato District Health Board, Hamilton 3204, New Zealand; 8 Department of Neurosurgery, Waikato District Health Board, Hamilton 3204, New Zealand

**Keywords:** p53 isoform, Δ133p53β, brain, metastasis, time to metastasis, receptor recycling

## Abstract

The Δ133p53β isoform is increased in many primary tumors and has many tumor-promoting properties that contribute to increased proliferation, migration and inflammation. Here we investigated whether Δ133p53β contributed to some of the most aggressive tumors that had metastasized to the brain. *Δ133p53β* mRNA expression was measured in lung, breast, melanoma, colorectal metastases and, where available, the matched primary tumor. The presence of *Δ133p53β* expression was associated with the time for the primary tumor to metastasize and overall survival once the tumor was detected in the brain. *Δ133p53β* was present in over 50% of lung, breast, melanoma and colorectal metastases to the brain. It was also increased in the brain metastases compared with the matched primary tumor. Brain metastases with *Δ133p53β* expressed were associated with a reduced time for the primary tumor to metastasize to the brain compared with tumors with no *Δ133p53β* expression. In-vitro-based analyses in Δ133p53β-expressing cells showed increased cancer-promoting proteins on the cell surface and increased downstream p-AKT and p-MAPK signaling. Δ133p53β-expressing cells also invaded more readily across a mock blood–brain barrier. Together these data suggested that Δ133p53β contributes to brain metastases by making cells more likely to invade the brain.

## 1. Introduction

Wild-type p53 is well known for its role in tumor suppression; however, *TP53* encodes at least 12 isoforms and some of these promote tumor progression. The Δ133p53 isoforms (Δ133p53α, Δ133p53β and Δ133p53γ) have been associated with cancer, with the Δ133p53β isoform being the most frequently increased in cancer, including brain, prostate, breast and colorectal tumors [1,2,3,4,5]. Δ133p53β-expressing tumors resist treatment, invade, recur and contain high numbers of tumor-supporting cells, such as tumor-associated macrophages (TAMs) and those that prevent cytotoxic T-cell anti-tumor responses [3,6]. Analysis of Δ133p53β was largely restricted to primary tumors. Considering cancer mortality is largely associated with metastases, with brain tumor metastases being a significant cause of mortality for 20–40% of cancer patients [7], Δ133p53β may be more prominent in more aggressive tumors, such as those that have metastasized to the brain. In an analysis of 18 brain tumor metastases from the breast, lung and colon, Δ133p53β protein aggregates were present in 72% of tumors, suggesting that Δ133p53β is present in the majority of brain metastases [8,9]. Here we investigated the role of Δ133p53 isoforms in a larger cohort of 140 brain metastases from tumor types most likely to go to the brain, including tumors from the breast, lung, colon and skin. We report that Δ133p53β was a key feature of brain metastases from all tumor types. Δ133p53β had properties that may make the tumors more likely to enter the brain, providing a possible explanation for why it was associated with a reduced time for primary tumors to spread to the brain.

## 2. Results

### 2.1. Δ133p53β Was Present in Many Brain Tumor Metastases

**Figure 1:**
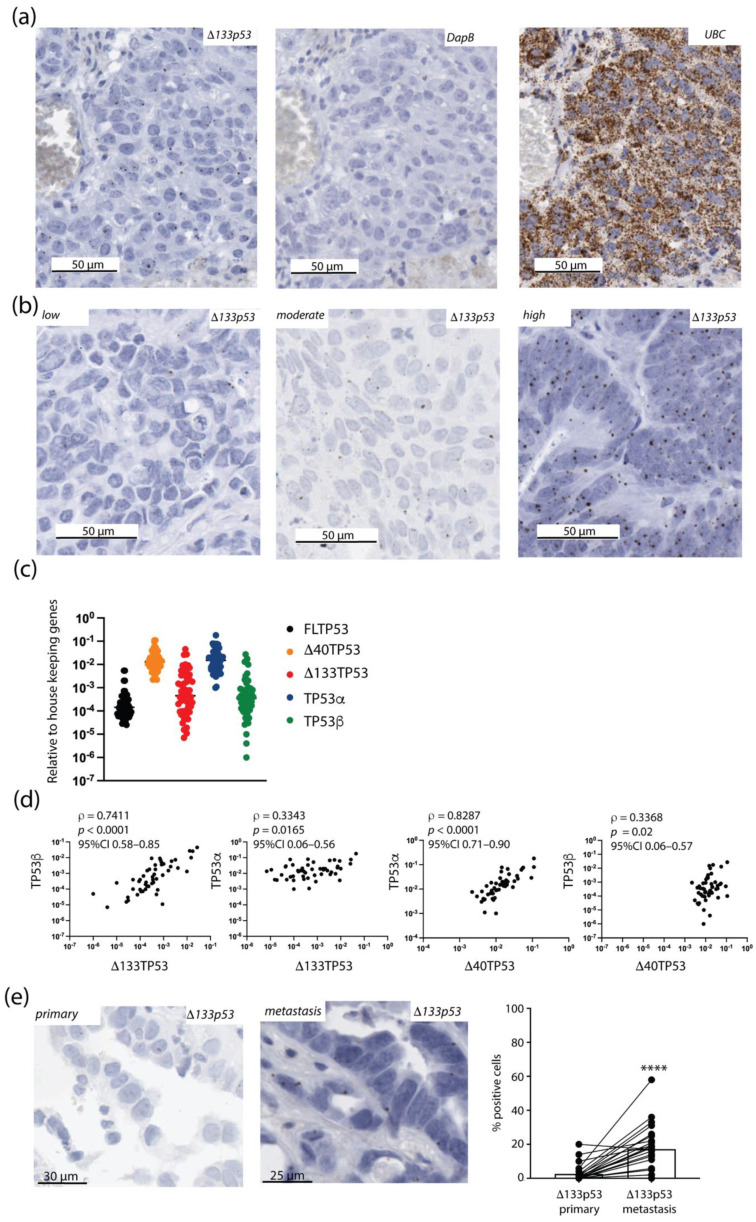
*Δ133p53β* mRNA is present in tumors that metastasized to the brain. (**a**) *Δ133p53β* mRNA expression in a brain metastasis using RNAscope alongside the *UBC*-positive and *DapB*-negative controls. (**b**) Representative images that highlight brain metastases with low, moderate and high *Δ133p53β* expression using RNAscope. (**c**) *TP53* transcripts were measured in brain tumor metastases with frozen tissue available using real-time quantitative PCR. Results: mean ± s.d. (**d**) Correlation between *TP53* transcripts to estimate individual isoforms. (**e**) Increased Δ133p53β mRNA expression in the brain metastasis compared with the matched primary tumor. Left: *Δ133p53β* expression in a matched primary and brain metastasis pair. Right: results from all tumors with matching primary and brain metastasis. Results: mean ± s.d. **** *p* < 0.0001. Black dots, individual results.

Breast, colorectal, lung and melanoma brain metastases (n = 140) were included in the study (Table 1). All tumors were subjected to RNAscope using a probe toward *Δ133p53β* mRNA (Figure 1 and Supplementary Figure S1) [2,3]. All tumor sections showed strong staining for *ubiquitin C* (*UBC*), except in areas with necrosis, indicating that each tumor had good RNA preservation (Figure 1a). Tumors were classified as having no (0%), low (2–10%), moderate (11–25%) or high (>25%) *Δ133p53β* mRNA expression based on the percentage of positive cells (Figure 1b). All tumors with low, moderate or high *Δ133p53β* were considered positive for *Δ133p53β* expression, provided the *DapB* negative control showed no staining, and all tumors with no *Δ133p53β* staining were considered negative for *Δ133p53β* mRNA expression.

Cases positive for *Δ133p53β* mRNA had positive cells spread throughout the malignant cells in the tissue section, suggesting that *Δ133p5*3*β* was commonly expressed. In breast metastases, 63% (*n* = 17/27) of tumors were positive for Δ133p53β, with positive tumors having between 2 and 57% positive cells (median, 16%). In colorectal metastases, 57% of positive cells (*n* = 12/21) of tumors were positive for Δ133p53β, with positive tumors having between 2 and 44% positive cells (median, 33.5%). In lung metastases, 61% (*n* = 28/46) of tumors were positive for *Δ133p5*3*β*, with positive tumors having between 2 and 57% positive cells (median, 12%). In melanoma metastases, 57% (*n* = 26/46) of tumors were positive for Δ133p53β, with positive tumors having between 2 and 50% positive cells (median, 13%).

Although the RNAcope probe best detects *Δ133p53β*, it may also detect other Δ133p53 isoforms. To confirm that *Δ133p53β* was increased in brain metastases, real-time quantitative PCR was used to identify all *full-length TP53* (*FLTP53*), *Δ40TP53* and *Δ133TP53* isoforms in 51 brain metastases with frozen tissue also available (Figure 1c). The *Δ133TP53* variant had a strong positive correlation with the β isoform (*TP53β*, ρ = 0.7411, *p* < 0.0001) and a weaker positive correction with the α isoform (*TP53α*, ρ = 0.3343, *p* = 0.0165; Figure 1d). A strong positive correlation was also found for *Δ40TP53* and *TP53α*, and a weaker correction was found between *Δ40TP53* and *TP53β* (Figure 1d). In brain metastases, *Δ133TP53β* and *Δ40TP53*α were the most prominent *TP53* transcripts.

### 2.2. Δ133p53β Was Increased in Metastases Compared with the Primary Tumor

The matched primary tumor was available for 24 brain tumor metastases. Using RNAscope, 11 (46%) primary tumors expressed *Δ133p*53*β* mRNA. Of the primary tumors with no *Δ133p53β* mRNA expression, 10 showed *Δ133p53β* mRNA expression in the metastases, suggesting that *Δ133p5*3*β* mRNA was increased in more aggressive tumors. Many primary tumors did not show *Δ133p53β* -positive staining across the entire tumor; instead, regions of positive staining were present. The percentage of positive cells were lower in primary tumors compared with the matching brain metastases (mean percentage of positive cells: 2.6 in primary tumors versus 17.8 for brain metastases, *p* < 0.0001, Figure 1e and Supplementary Figure S2).

### 2.3. Δ133p53β Was Associated with a Reduced Time to Metastasize to the Brain

**Figure 2:**
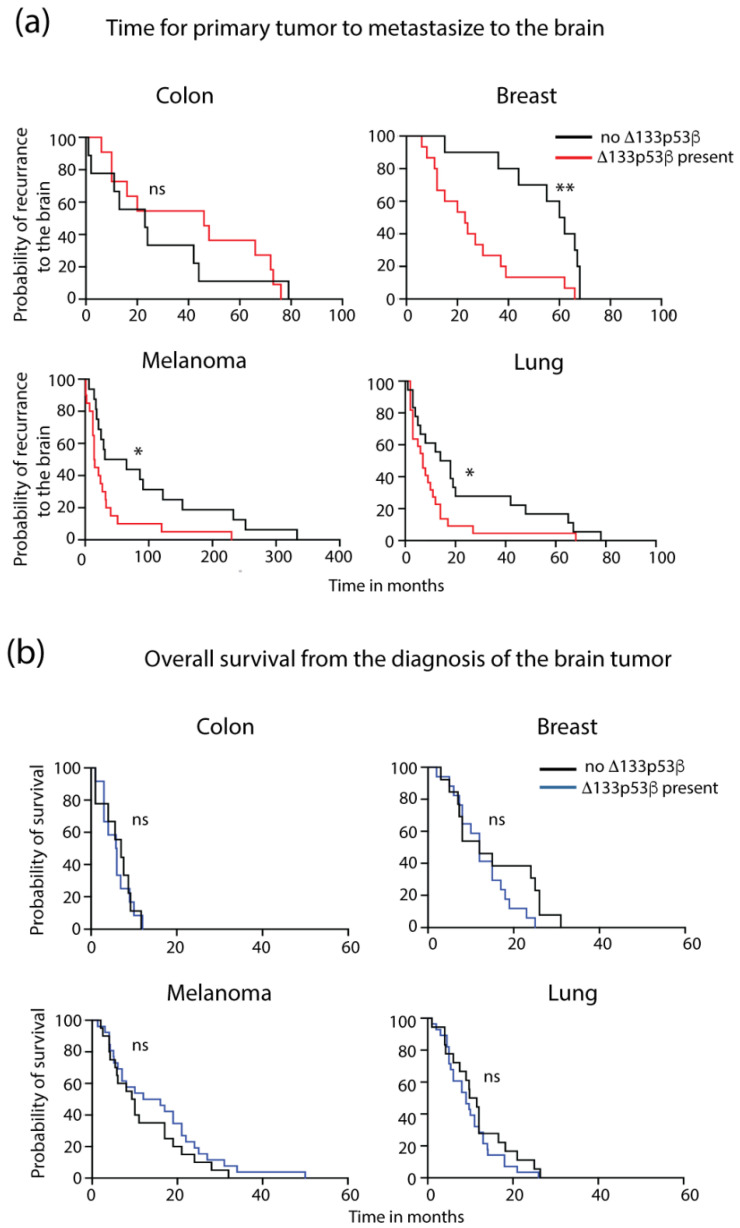
Patient outcome based on tumor *Δ133p53β* expression. (**a**) Time for the primary tumor to progress to the brain. (**b**) Overall survival time once the tumor was diagnosed in the brain. Δ133p53β was measured using RNAscope in breast, melanoma, lung and colorectal brain metastases and the patient outcomes were compared between those with *Δ133p5*3*β*-expressing (high, medium, or low) tumors and those with no *Δ133p53β* expression. * *p* < 0.05; ** *p* < 0.01; ns, not significant.

The finding of increased *Δ133p53β* in the metastasis compared with the primary tumor was consistent with a role for Δ133p53β in more aggressive tumors. To investigate this further, a positive or negative Δ133p53β status in the brain metastasis was compared with the time for the primary tumor to metastasize to the brain and the overall patient survival once the tumor was diagnosed in the brain. A significant reduction in the time for the primary tumor to metastasize to the brain was found for breast (61 versus 23 months, *p* = 0.0028; ratio 2.652, 95% CI 1.91–5.904), melanoma (48 versus 14.5 months, *p* = 0.0114; ratio 3.319, 95% CI 1.715–6.388) and lung (16 versus 7 months, *p* = 0.0466; ratio 2.286, 95% CI 1.226–4.261) tumors that were positive for Δ133p53β compared with negative tumors (Figure 2a). No significant difference in time was found for positive colorectal tumors (46 versus 23 months, *p* = 0.559; ratio 1.186, 95% CI 0.4999–2.816) compared with Δ133p53β-negative tumors). No difference in overall patient survival from when the tumor was diagnosed in the brain was found between Δ133p53β-positive and -negative tumors across all tumor types (*p* > 0.05, Figure 2b). These findings suggested that increased Δ133p53β in brain metastasis identified tumors that spread faster to the brain, but once the tumor was in the brain, Δ133p53β did not affect overall patient survival.

**Figure 3:**
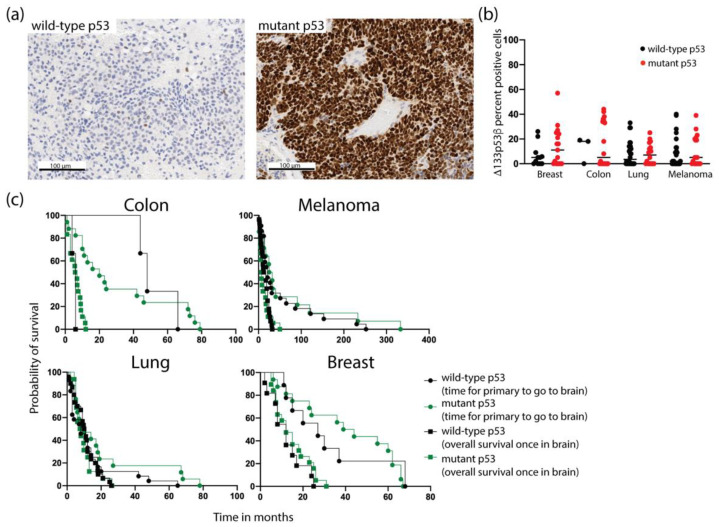
Correlation between mutant p53 and Δ133p53β expression in brain metastases. (**a**) Mutant p53 was estimated as intense staining in over 80% of tumor cells using immunohistochemistry. (**b**) Across brain metastases, mutant p53 was not correlated with Δ133p53β mRNA expression from RNAscope analyses. (**c**) Mutant p53 was not associated with altered patient progression time for the primary tumor to metastasize to the brain or with overall survival when the tumor was diagnosed in the brain across different tumor types.

We next investigated whether there was an association between *Δ133p5*3*β* expression and mutant p53, as mutant p53 promotes metastasis [10]. Immunohistochemistry was used as a surrogate for identifying tumors with mutant p53 based on mutations in p53 increasing the stability of the p53 protein. Strong widespread expression of p53 consistent with mutant p53 was assigned to 56% of breast metastases (*n* = 15/27), 86% of colorectal metastases (*n* = 18/21), 39% of lung metastases (*n* = 18/46) and 41% of melanoma metastases (*n* = 19/46). All tumors designated as mutant for p53 showed strong nuclear staining in almost 100% of the tumor cells (Figure 3a). There was no correlation between the presence of mutant p53 and *Δ133p53β* using RNAscope (Figure 3b). Mutant p53 in the brain metastases was not associated with reduced time for the primary tumor to metastasize to the brain or overall patient survival once the tumor was detected in the brain (Figure 3c).

### 2.4. RNA Sequencing Showed That Δ133p53β Was Associated with Altered Gene Expression

**Figure 4:**
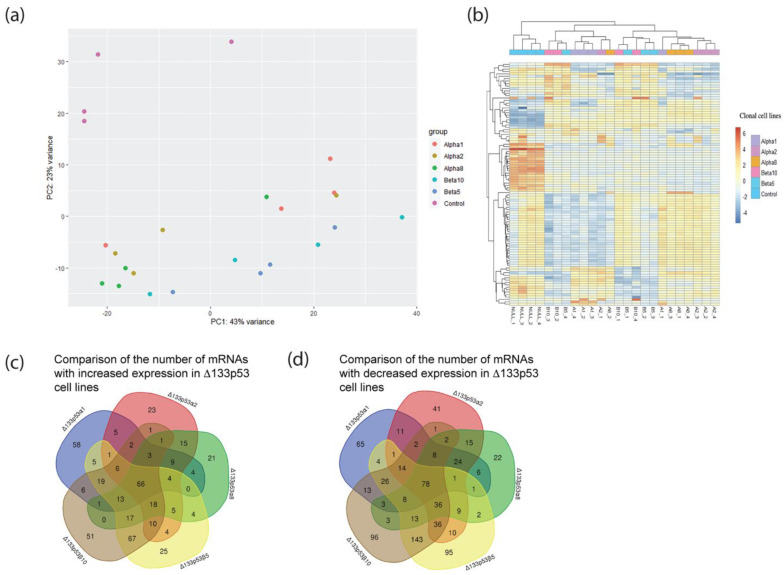
Introduction of Δ133p53α and Δ133p53β to H1299 cells resulted in distinct but overall subtle changes in gene expression. (**a**) Principal component analysis showing cell lines expressing Δ133p53α or Δ133p53β clustered distinctly from control cells without Δ133p53 isoforms. (**b**) Top 200 most variable genes clustered control cells together, but not Δ133p53α or Δ133p53β cell lines. (**c**) Venn diagram of transcripts increased in Δ133p53α and/or Δ133p53β cell lines. (**d**) Venn diagram of transcripts decreased in Δ133p53α and/or Δ133p53β cell lines. Alpha 1, Δ133p53α1; Alpha 2, Δ133p53α2; Alpha 8, Δ133p53α8; Beta 5, Δ133p53β5; Beta 10, Δ133p53β10; Control, H1299 p53 null.

To provide more insight into how Δ133p53β contributes to metastasis, RNA sequencing (RNAseq) was performed on five clonal cell lines that stably expressed Δ133p53α (Δ133p53α1, Δ133p53α2 and Δ133p53α8) or Δ133p53β (Δ133p53β5 and Δ133p53β10), which were cell lines created by adding individual Δ133p53 isoforms to the p53 null cell line H1299 [2]. H1299 vector control cells were included as a control (Figure 4). A principal component analysis (PCA) and unsupervised hierarchical clustering analysis revealed the p53 null cell line clustered distinctly away from the Δ133p53α and Δ133p53β cell lines. Differences between the Δ133p53α and Δ133p53β cell lines were subtle, though they were more evident in the PCA compared with the clustering analysis (Figure 4a,b). No biological processes were enriched in differentially expressed genes using an FDR cutoff of <0.05.

**Figure 5:**
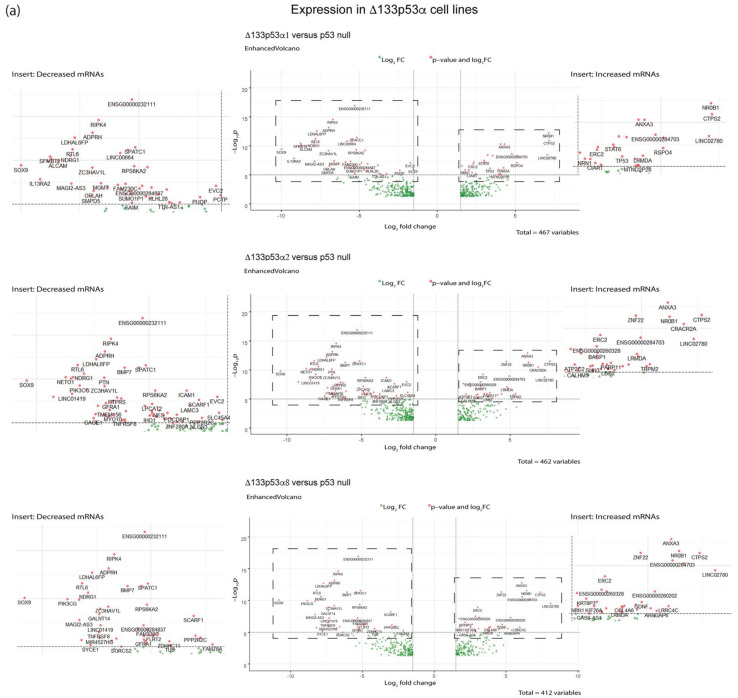

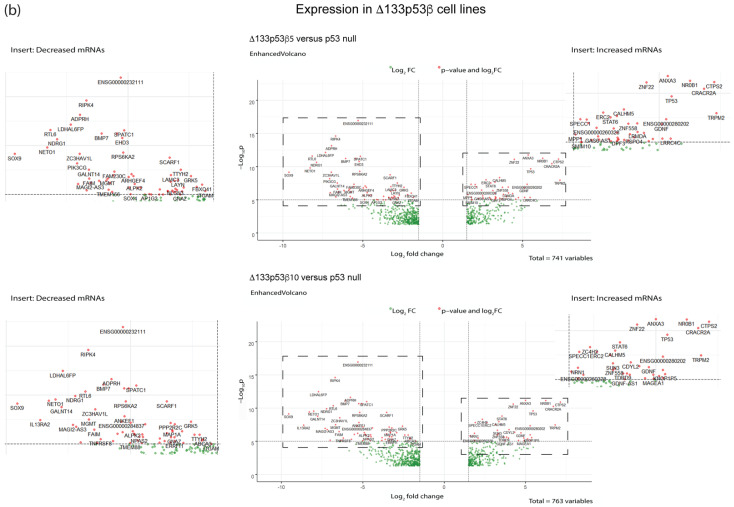
Volcano plots of gene transcripts altered in Δ133p53α or Δ133p53β cells compared with the control (p53 null). (**a**) Transcripts increased and decreased in Δ133p53α cell lines and (**b**) transcripts increased and decreased in Δ133p53β cell lines. Transcripts highlighted in red were considered statistically differentially expressed (criteria of false discovery rate (FDR) < 0.05 and log_2_ fold change of ±1.5 (FC ± 1.5)). Boxed areas shown as inserts to the left and right.

An analysis of individual transcripts showed that consistent transcripts were increased or decreased in Δ133p53β and Δ133p53α cells (Figure 5). *SOX9*, *NDGR1*, *RIP4*, *RTL6*, *LDHAL6FP*, *ADRPH* and *SPATC1* were decreased in all Δ133p53α and Δ133p53β cell lines and *MGMT* was decreased in Δ133p53β cell lines. *ANAX3* and *CTP32* were increased in all Δ133p53α and Δ133p53β cell lines, with *STAT6*, *CALHM5*, *NR0B1*, *TPM2*, *CRACR2A*, *GDNF* and *ZNF22* increased in Δ133p53β cell lines and *ERC2* increased in Δ133p53α cell lines.

Transcripts that encoded the tumor suppressors NDGR1 and RIP4 were decreased and transcripts that encoded the tumor-promoting proteins GDNF, ZNF22 and ANXA3 were increased in Δ133p53 cells, particularly Δ133p53β, which is consistent with a role for Δ133p53β in promoting cancer; however, some results were inconsistent with a role for Δ133p53β in promoting cancer, with increased expression of the tumor suppressor *TPM2* and decreased expression of *SOX9,* which was described as having both tumor suppressor and tumor-promoting properties [11,12,13,14,15,16,17,18].

**Figure 6:**
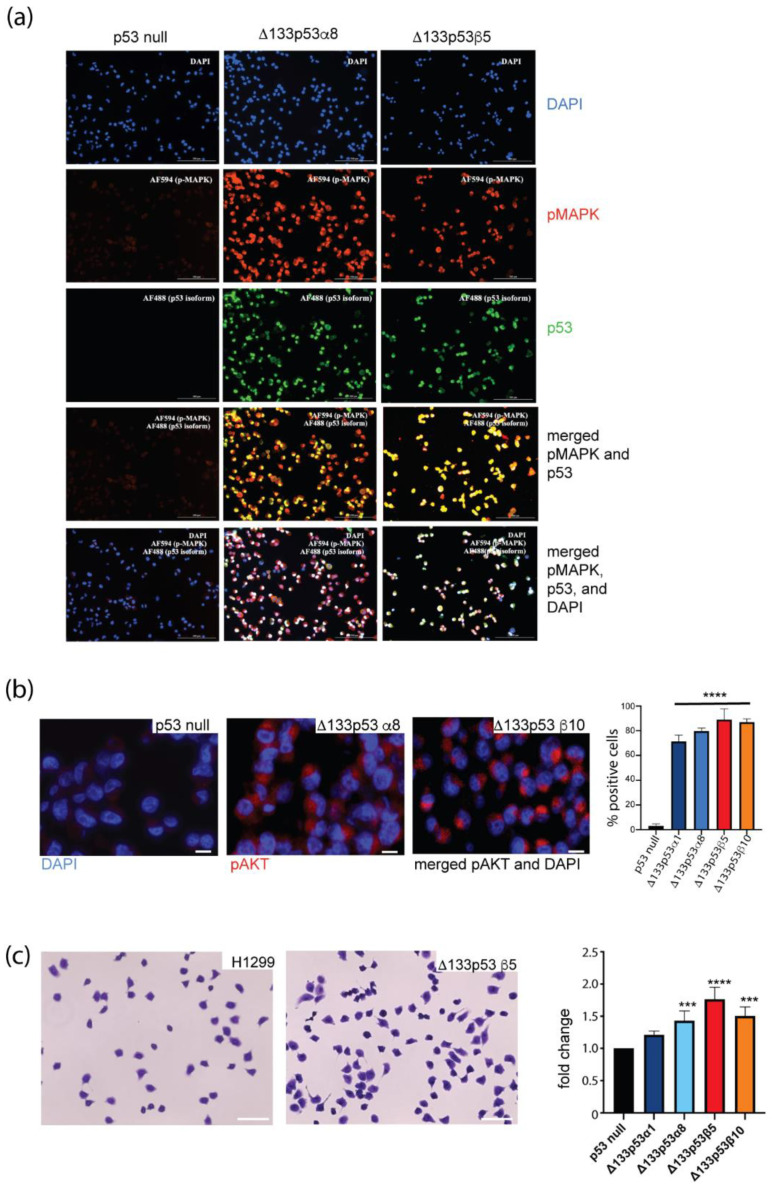
Cancer-promoting pathways increased with Δ133p53α and Δ133p53β. (**a**) Increased p-MAPK (scale bar 200 µm) and (**b**) p-AKT (scale bar 25 µm) were evident when using immunofluorescence in Δ133p53α and Δ133p53β cells. (**c**) Δ133p53-expressing cells were more likely to move through an in vitro blood–brain barrier. Crystal violet staining (left, scale bar 100 µm) and quantification (right) of cells that moved through the barrier. Results: mean ± s.d. from at least three replicates. *** *p* < 0.001, **** *p* < 0.0001.

The RNAseq analysis showed subtle changes in gene expression with the introduction of Δ133p53α or Δ133p53β. To determine whether growth-promoting signaling was increased overall in Δ133p53 cells, immunofluorescence for p-MAPK, p-AKT and major downstream effector pathways in oncogenic signaling was performed. Δ133p53 cells showed marked increases in p-MAPK- and p-AKT-positive cells (Figure 6a,b).

These results suggested that despite subtle gene expression differences in Δ133p53 cells, the oncogenic signaling pathways were activated.

After confirming that p-MAPK and p-AKT signaling were increased in Δ133p53α and Δ133p53β cells, we next investigated whether Δ133p53 cells had functions that were consistent with promoting cancer, including brain metastasis. Using an in vitro blood–brain barrier containing human microvascular endothelial cells (HBMECs) on the apical surface and astrocytes on the basolateral surface of a transwell insert [19], Δ133p53β cell lines and one Δ133p53α cell line showed greater invasion across the barrier compared with control cells (Figure 6c).

These findings suggested that increased Δ133p53 makes tumors more invasive and more likely to enter the brain.

### 2.5. Δ133p53β Had Increased Cancer-Promoting Proteins on the Cell Surface

**Figure 7:**
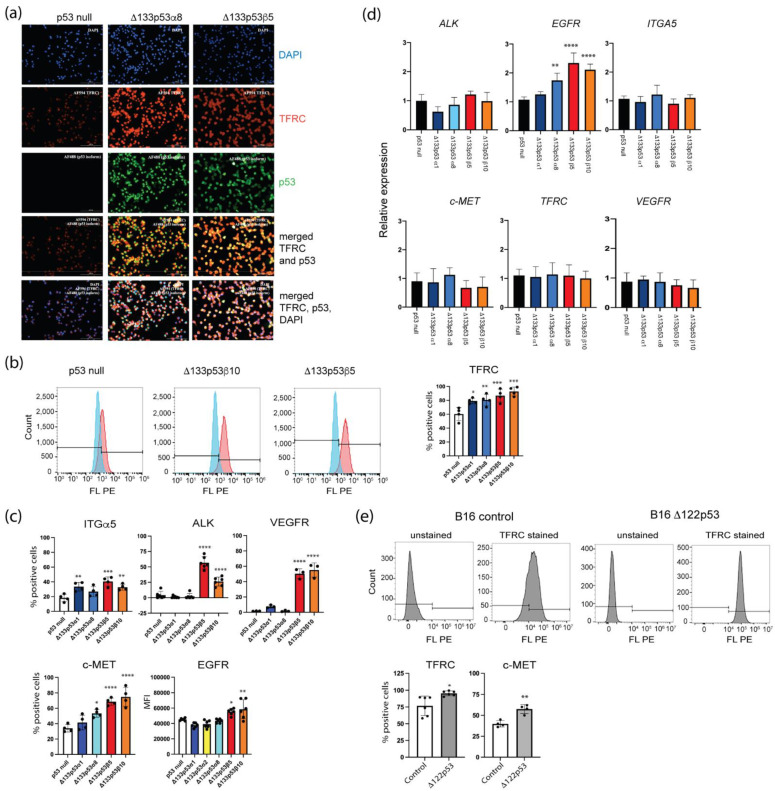
Cancer-promoting proteins increased on the Δ133p53α and Δ133p53β cell surface. (**a**) Increased TFRC staining in Δ133p53 cells using immunofluorescence. Scale bar 200 µm. (**b**) Flow cytometry to identify the transferrin receptor (TFRC) on the cell surface. Left: histogram for TFRC staining, with unstained cells in blue and stained cells in red. Right: quantification of TFRC amongst Δ133p53α and Δ133p53β cell lines. (**c**) Flow cytometry to compare the presence of other cancer-promoting proteins on the Δ133p53 cell surface. (**d**) Most proteins that increased on the 133p53α and Δ133p53β surface were not explained by altered gene expression using real-time quantitative PCR. (**e**) Flow cytometry to identify TFRC and c-Met on the cell surface of murine B16 melanoma cells expressing a mimic of Δ133p53α (Δ122p53) or the control vector (B16 control). Top: histogram for TFRC staining. Bottom: quantification of TFRC and C-Met. Results are relative to the expression in the p53 null cell line. Results: mean ± s.d. from at least three replicates. * *p* < 0.05, ** *p* < 0.01, *** *p* < 0.001, **** *p* < 0.0001. Black dots, individual results.

The increased p-AKT and p-MAPK signaling in Δ133p53 cells suggested that Δ133p53 may be associated with increased growth signaling from the cell surface. To determine whether cancer-promoting proteins were increased on the Δ133p53 cell surface, proto-oncogenes or proteins induced by proto-oncogenes in lung cancer, including c-MET, VEGFR, ITGα5, EGFR, TFRC and ALK, were measured on the cell surface of Δ133p53α and Δ133p53β cell lines using flow cytometry (Figure 7a–c). All proteins were increased on Δ133p53β cells, and c-MET, ITGα5 and TFRC were also increased on Δ133p53α cells. Increased TFRC in Δ133p53α and Δ133p53β cells was also confirmed using immunofluorescence (Figure 7a). With the exception of EGFR, the increased expression of the corresponding genes did not explain the increase in cell surface proteins, as was evident using TaqMan qPCR assays (Figure 7d).

Whether Δ133p53 isoforms could increase cancer-promoting proteins on the surface was tested further using B16 murine melanoma cell lines expressing wild-type p53 or Δ122p53 (a mimic of Δ133p53α function) [20]. Flow cytometry revealed increased c-Met and TRFC in Δ122p53 cells compared with the control (Figure 7e).

These findings suggested that some cancer-promoting properties of Δ133p53β and Δ133p53α occur by increasing growth-promoting receptors on the cell surface.

### 2.6. Increased Cell Surface Δ133p53β Protein Phenotype Could Be Used to Target Δ133p53β Cells

**Figure 8:**
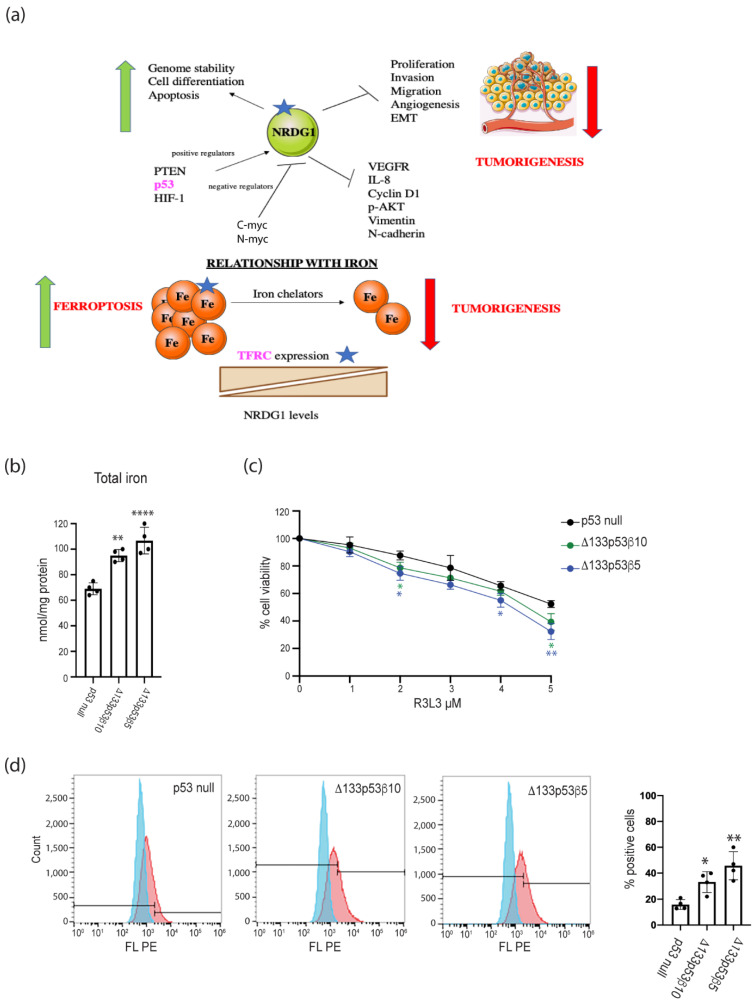
Increase in cell surface proteins can be used to target Δ133p53β cells. (**a**) Increased TFRC and decreased NRDG1 promoted tumorigenesis, including intracellular iron concentrations. Green arrows, pathways increased; red arrows, pathways decreased. Blue stars, potential therapeutic targets to affect Δ133p53β function. (**b**) Comparison of total intracellular iron concentrations. (**c**) Comparison of cell viability following ferroptosis induction with R3L3. (**d**) Left: histograms for the measurement of cellular lipid peroxidases (blue signifies unstained cells, red signifies cells incubated with C-11 BODIPY) using flow cytometry. Right: comparison of the percentages of C11-BODIPY-positive cells. Results: mean ± s.d. from at least three replicates. * *p* < 0.05, ** *p* < 0.01, **** *p* < 0.0001. The top right image in (**a**) is from Servier Medical ART: SMART.

The decreased expression of *NDGR1* and increased TFRC on the cell surface were consistent with the role of Δ133p53β in promoting cancer (Figure 8a). Since both TFRC and NRDG1 also have opposing functions in iron regulation and increased TFRC can reduce cell viability upon ferroptosis induction with the GPX inhibitor (1S,3R)-RSL3 (RSL3) [21], we investigated whether the increased TFRC on the cell surface would make Δ133p53 cells more susceptible to iron-related cell death. Consistent with Δ133p53β having increased TFRC expression, Δ133p53β cell lines had increased intracellular total iron concentrations relative to the total protein (Figure 8b). Upon ferroptosis induction with RSL3, Δ133p53β cells showed reduced viability and increased cellular lipid peroxides (Figure 8c,d).

These results suggested that Δ133p53β cells are more susceptible to ferroptosis and the increased proteins on the cell surface could be manipulated to target Δ133p53β cells.

## 3. Discussion

Δ133p53β is present in many aggressive primary tumors and here we extended this to some of the most aggressive tumors that metastasize to the brain. Δ133p53 was present across tumor types and increased in metastasized tumors compared with primary tumors. Δ133p53β was associated with a reduced time for the primary tumor to metastasize to the brain. In addition, the introduction of Δ133p53β in a lung cell line increased migration across a transwell assay that mimicked aspects of the blood–brain barrier.

Δ133p53β pro-migratory properties may make tumors more likely to enter the brain, as was evident with Δ133p53 tumors showing a reduced time to metastasize to the brain. In primary breast, colon, brain and prostate tumors Δ133p53 isoforms, with a predominance for the Δ133p53β isoform, are associated with poorer clinical outcomes, which is consistent with Δ133p53β being a characteristic of aggressive tumors [2,3,5,22]. In breast cancer, Δ133p53β increased the risk of recurrence and death and was the most significant independent predictor of outcome in tumors that would otherwise be predicted to have a good prognosis [1]. In prostate cancers, Δ133p53β alone predicted aggressive disease with 88% accuracy [2]. In breast, colon and prostate cancer, the association of Δ133p53β with increased risk of recurrence suggests Δ133p53β increases the likelihood of invasion from the primary site [2,3,5,22]. In in vitro corroboration studies, Δ133p53β and a murine mimic of Δ133p53 (Δ122p53) increased the migration potential in cells with wild-type and mutant p53 and in those with otherwise poor invasive properties [1,5]. In an orthotopic transplantation model, Δ133p53 has three-fold higher expression in a highly metastatic cell line with reduced time to metastasis [23].

Δ133p53β produces highly invasive cells by promoting the loss of adhesive structures and a rounded amoeboid morphology [1]. The findings from this study also found enhanced cancer-promoting functions with Δ133p53β compared with Δ133p53α. Δ133p53β clonal cell lines showed increased migration across a transwell containing endothelial and astrocytes to mimic the more restrictive blood–brain barrier, suggesting that Δ133p53β would enhance movement into the brain. In addition, many well-characterized tumor-promoting proteins, including those with roles in metastasis, were increased on the cell surface, including c-MET, EGFR, VEGFR, ALK and ITGα5.

The relatively subtle changes in gene expression compared with the proportion of cell surface proteins studied that were found to have increased on the Δ133p53β cell surface suggested that a larger pan-proteomic-based approach may identify more pathways that are associated with Δ133p53β function. Increased proteins on the cell surface without changes in the expression of the corresponding genes were reported for mutant p53. Mutant p53 was proposed to promote enhanced receptor recycling of c-MET and ITGα5 to the cell surface in mutant p53 cancers, leading to increased cell surface protein expression, downstream signaling and effector functions of enhanced scattering and invasion [24,25]. Other evidence supports a role for Δ133p53α and Δ133p53β in increasing cell surface proteins; GLUT1 was increased in Δ133p53α-expressing MCF7 cells; and in the murine model to investigate Δ133p53α function, Δ122p53-positive peripheral blood mononuclear cells showed increased alpha-enolase on the cell surface and the associated plasminogen activation [26,27].

Δ133p53α and Δ133p53β isoforms may share with mutant p53 an ability to enhance the return of cancer-promoting proteins to the cell surface, suggesting oncogenic variants of p53 may promote metastasis by enhancing signaling from the cell surface. The markedly high p-AKT and p-MAPK staining in Δ133p53α and Δ133p53β cells are consistent with multiple cancer-promoting proteins being increased on the Δ133p53 cell surface. The altered cell surface protein phenotype could be manipulated as a means to target Δ133p53α and Δ133p53β cells, as demonstrated here with increased TFRC, which may have been aided by a corresponding decrease in NDGR1, leading to an increased susceptibility to induced ferroptosis.

Increased expression of cancer-promoting proteins on the Δ133p53α cell surface has not been universally found, with reduced ITGα5 found in MCF10A cells [28]. The overall p53 and p53 family environment may be important for determining the overall effects of Δ133p53α and Δ133p53β on the cell surface, with other studies finding the binding of mutant p53 or Δ133p53 to p63 or p53α responsible for the changes to individual cell surface proteins. Other p53 proteins were not present in H1299 expressing cells used here, but brain metastases did have multiple p53 moieties. Unlike primary brain tumors where Δ133p53 was almost mutually exclusive with mutant p53, many brain metastases harbor both mutant p53 and Δ133p53, suggesting that both may contribute to brain metastases. Δ133p53 was not the only *TP53* isoform increased in brain metastases, with *Δ40p53α* also showing a higher expression. The cancer-promoting functions of Δ40p53 are less thoroughly characterized. However, in response to DNA damage, Δ40p53 inhibited apoptosis [29]. 

How Δ133p53 isoforms are elevated in cancers is unclear, but possibilities include hypoxia and infection [2,3,30,31]. Δ133p53 contributes to treatment resistance, and investigations of other p53 isoforms found increased expression in response to DNA-damaging cancer treatments, which suggests that Δ133p53 may also increase with treatment. Increased Δ133p53 with chemotherapy and downstream resistance to treatment offers an explanation for why Δ133p53 is elevated in brain metastases compared with the primary tumor [3,32,33].

The limitations of this study were those specific to studying brain tumors, where only tumors that were removed, and potentially not very aggressive inoperable tumors, were represented in the cohort. The survival analyses were not adjusted for confounding factors; these are numerous for patients with brain metastases, and thus, a much larger cohort size is required.

Overall, the results from this study indicate that Δ133p53β is increased in brain metastases. Δ133p53β may allow tumors to more readily enter the brain, reducing the time for primary tumors to metastasize to the brain.

## 4. Materials and Methods

### 4.1. Human Tumors

Brain tumor metastases from primary lung (*n* = 46), breast (*n* = 27), melanoma (*n* = 46) and colorectum (*n* = 21) tumors were selected from those collected at Dunedin and Hamilton hospitals. Following surgery, the tumors were either fixed in 10% neutral buffered formalin or snap-frozen in liquid nitrogen and stored at −80 °C. Fixed tissue was processed into paraffin wax and embedded. Formalin-fixed paraffin-embedded tumors were cut on a microtome into either 4 µm (immunohistochemistry) or 5 µm (RNAscope) tissue sections that were placed onto coated slides (Leica Bond, Leica Biosystems, Wetzlar, Germany). Paraffin-embedded formalin-fixed tissues were available for all tumors and additional frozen tissue was also available for 51 tumors. Matching primary tumors were available for 24 cases (7 breast, 6 colorectal, 7 lung and 3 melanoma). The clinicopathological data associated with all samples are outlined in Table 1. The inclusion criteria were brain tumor metastases with tumor tissue removed between 2010 and 2016 diagnosed as breast, lung, colorectal or lung metastases with only tissue from the first brain tumor metastasis analyzed in cases with recurrent brain tumors excised. Ethical approval (reference LRS/10/09/037 and MEC/08/02/061) was obtained in New Zealand and all procedures followed institutional guidelines. All individuals provided written informed consent.

### 4.2. RNAscope

Probes included a custom probe to the unique region of *∆133TP53* and *TP53β* made by Advanced Cell Diagnostics named *∆133TP53* and described elsewhere [2,3], as well as the probes *ubiquitin C* (*UBC*, positive control) and *DapB* (negative control, Advanced Cell Diagnostics, Newark, CA, USA). Formalin-fixed paraffin-embedded tumors or cell clots were cut into 5 µm sections. The RNAscope method used the manual assay method with Protease Plus reagent for protein digestion and the 2.5HD reagent kit brown according to the manufacturer’s instructions. Following the addition of DAB to the *∆133TP53* and *DapB* assays, DAB enhancer was added (Leica Biosystems, Wetzlar, Germany). Positive cells and the number of dots per positive cell were identified using the Aperio Scancope CS digital pathology system and quantified using the Aperio RNA ISH Algorithm (Aperio, Vista, CA, USA). The slides were evaluated by two blinded examiners. The percentage of positive cells out of the total cell number was measured. Ten fields (×400 magnification) were chosen at random and the percentage of positive cells out of the total cells counted. Tumors with no positive cells in the 10 random fields were examined over the entire tissue section for the presence of positive cells.

### 4.3. RNA, cDNA and Gene Expression Assays

Total RNA was prepared using a PureLink™ RNA Mini Kit (Invitrogen, Carlsbad, CA, USA); this extracted RNA was reverse-transcribed and then converted to cDNA using qScript cDNA SuperMix (Quantabio, Beverly, MA, USA). The protocol included DNase I, Amp Grade (ThermoFisher Scientific, Waltham, MA, USA). Four to six repeats per cell line were performed. Gene expression was quantified in triplicate from single cDNA preparations using the LightCycler 480 (LC480) (Roche, Basel, Switzerland). Relative gene expression was calculated using the double delta Ct (ΔΔCT) method and qbase^+^ software version 3.0 [34]. Expression assays for *Δ133TP53* isoforms were performed as described previously [3,35]. TaqMan expression assays were used to measure the following (Thermofisher Scientific, Waltham, MA, USA): ACVRL1 (ALK, Hs06638255_s1), EGFR (Hs01076090_m1), ITGA5 (Hs01547673_m1), c-MET (Hs01565584_m1), TFRC (Hs00951083_m1) and VEGFR (Hs01052961_m1). Tumour protein, translationally-controlled 1 (TPT1, Hs02621289_g1), glyceraldehyde 3-phosphate dehydrogenase (GADPH, Hs03929097_g1) and eukaryotic translation elongation factor 1-alpha 1 (EEF1A1, Hs00265885_g1) were used as reference genes alongside a non-template control (NTC). Four to six repeats per cell line were performed. Gene expression was quantified in triplicate from single cDNA preparations using the LightCycler 480 (LC480) (Roche, Basel, Switzerland). Relative gene expression was calculated using the double delta Ct (ΔΔCT) method and qbase^+^ software [34].

### 4.4. Cell Lines

Stable clonal cell lines expressing different Δ133p53 isoforms derived from H1299 cells, namely, Δ133p53α (α1, α2 and α8) and Δ133p53β (β5 and β10), and H1299 vector-only control cells (p53 null) were those described previously [2]. B16F1 mouse melanoma cell lines expressing the vector control (B16 control) or a murine protein similar to Δ133p53α (Δ122p53) were also used in the flow cytometry experiments as described previously [20]. 

### 4.5. RNAseq

Library generation (DNBSEQ Eukaryotic Strand-Specific mRNA library) and RNA sequencing (read length 100 base pair reads and the DNBseq platform) were performed by BGI Genomics in Hong Kong, China. Raw data was subject to quality control using Fast QC version 0.11.9. Adaptor sequences and low-quality sequences (Phred score < 30) were trimmed using Cutadapt version 2.6 [36]. Reads were aligned against the human reference genome (GRCh38) using HISAT2 version 2.1.0 and annotated with GRCh38.107.gtf from Ensembl [37,38]. Aligned reads were counted by exon and summarized by gene using featureCounts version v1.5.3 and the counts were normalized for differential gene expression using Wald Test from DESeq2 package (version 1.30.1) performed locally on R (version 4.0.2) and RStudio™ (version 1.3.1093) [39,40,41]. The *p*-values were adjusted for multiple testing using the Benjamini–Hochberg correction. Genes were considered statistically differentially expressed if the criteria of false discovery rate (FDR) < 0.05 and log_2_ fold change of ±1.5 (FC ± 1.5) were satisfied (Supplementary File S1).

### 4.6. In Vitro Blood–Brain Barrier

An in vitro blood–brain barrier was established using a co-culture cell model that contained human primary astrocytes and human microvascular endothelial cells (HBMECs, ThermoFisher Scientific, Waltham, MA, USA). Cells were initially cultured using a specialized cell culture medium as per the manufacturer’s instructions (HBMECs, Medium 131 with microvascular growth supplement and Gibco Astrocyte Medium for human astrocytes). Cells were maintained at 37 °C in 5% CO_2_ and used before passage 6. Once confluent, the cells were harvested for the transwell assay using Corning Transwell-Col collagen-coated membrane inserts, 12 mm, 0.4 µm pore PTFE membrane inserts (Merck KGaA, Darmstadt, Germany). Astrocytes (3.5 × 10^5^ cells) were seeded onto the basolateral transwell insert coated with poly-L-Lysine (Millipore, Sigma-Aldrich, St. Louis, MO, USA). Three to four days later, HBMECs (8 × 10^4^ cells) were seeded onto the apical side of the transwell insert. The supplemented astrocyte media was placed in the basal chamber and the HBMEC media was placed in the apical chamber. Once the astrocytes and HBMECs reached 90% confluency, the transepithelial resistance (TEER) was measured to determine the integrity of the barrier using the Epithelial Volt/Ohm (TEER) meter (World Precision Instruments, Sarasota, FL, USA). Once the TEER was ≥45 Ω/cm^2^, the *∆133TP53* or control p53 null cells (1.25 × 10^4^ cells) were added, and 24 h later, the insert was removed and the cells on the bottom of the 12-well plate were fixed with 4% paraformaldehyde and stained with 3% crystal violet. The plates were then imaged using an inverted research microscope and all cells were counted. Data are presented as the fold change in the total number of cells compared with the control cells. Results: mean ± s.d. from at least three replicates. * *p* < 0.05, ** *p* < 0.01, *** *p* < 0.001, and **** *p* < 0.0001.

### 4.7. Immunohistochemistry and Immunofluorescence

Four-micrometer sections from formalin-fixed paraffin-embedded tissues were used for IHC. For IHC, p53 staining used the D07 clone from Cell Marque (Rocklin, CA, USA) and the Bond RX system (Leica Biosystems, Wetzlar, Germany) with the Bond Refine Detection kit. Stained cells were identified using the Aperio Scancope CS digital pathology system (Leica Biosystems, Wetzlar, Germany). The slides were evaluated by two blinded examiners. A tumor was considered p53 mutant positive if 80% of the tumor was positive for nuclear p53 staining [42,43,44].

For immunofluorescence analyses, Δ133p53α8 and Δ133p53β5 clonal cell lines were grown on coverslips (Deckglaser, 13 mm diameter) that had been autoclaved and coated with poly-L-lysine. After 16 h, the cells were fixed in 4% paraformaldehyde and stained using antibodies to the transferrin receptor (EPR20584, Abcam Cambridge, UK) and p53 (PAb241, ThermoFisher Scientific, Waltham, MA, USA, for Δ133p53α cells and DO-11 Bio-Rad Laboratories, Hercules, CA, USA, for Δ133p53β cells), and subsequent staining with secondary antibodies (Alexa Fluor 488 for p53 antibodies and Alexa Fluor 594 for TFRC; ThermoFisher Scientific, Waltham, MA, USA). The coverslips were incubated with DAPI (Spectral DAPI, Akoya Biosciences, Marlborough, MA, USA) and mounted on slides with Fluromount-G Mounting Medium (ThermoFisher Scientific, Waltham, MA, USA). The slides were visualized using the Lionheart FX automated microscope (BioTek, Winooski, Vermont, USA) and Biotex Gen5 Data Analysis Software (Agilent Technologies, Santa Clara, CA, USA).

### 4.8. Flow Cytometry for Cell Surface Proteins

Δ133p53α, Δ133p53β and control cells were cultured in RPMI 1640 media supplemented with 10% FCS. Cells were harvested using cell scrapers and 1 × 10^6^ cells were stained with LIVE/DEAD Fixable Yellow stain (ThermoFisher Scientific, Waltham, MA, USA) to identify the live cells. Cells were either left (unstained) or the cell surface was labeled with antibodies against TFRC (MEM-75 directly conjugated to PE, Abcam, Cambridge, UK), VEGFR1 (NB600-1006APCCY7, Novus Biologicals-Centennial, Englewood, CO, USA), ALK1 (MM0015-8G33, Abcam, Cambridge, UK), ITGα5 (P1D6, Abcam, Cambridge, UK), EGFR (AY13 APC conjugated, Biolegend, PerkinElmer, Waltham, MA, USA) and c-MET (243 PE-conjugated). For unconjugated antibodies, the following secondary was used: goat-anti mouse Alexa Fluor 488 (ThermoFisher Scientific, Waltham, MA, USA). B16 control and B16 Δ122p53 cells were cultured in DMEM media supplemented with 10% FCS and processed as described for Δ133p53 cell lines. Cells were either left unstained or the cell surface was labeled with antibodies against c-MET (EPR22436-24, Abcam, Cambridge, UK) or TFRC (EPR20584, Abcam, Cambridge, UK). The following secondary was used: PE donkey anti-rabbit (BioLegend, San Diego, CA, USA).

Unstained and stained cells were measured on a Gallios flow cytometer with Kaluza software version 2.2 (Beckman Coulter, Indianapolis, IN, USA). The percentage of positive cells was calculated using FLOWJO software version 10.6.2 (BD Biosciences, Franklin Lakes, NJ, USA). The percentage of positive cells was compared between Δ133p53 and control cells.

### 4.9. Total Iron Measurement

Measurement of total intracellular iron followed a published protocol with modifications [45]. The Iron Assay Kit (Sigma-Aldrich, St. Louis, MO, USA) and Pierce BCA Protein Assay Kit-Reducing Agent Compatible (ThermoFisher Scientific, Waltham, MA, USA) were used according to the manufacturer’s instructions.

### 4.10. Cell Viability Measurement

A total of 10,000 cells per well were seeded in 96-well plates and treated with RAS-selective lethal 3 (RSL3, 0–5 µM, MedChemExpress, Monmouth Junction, NJ, USA) for 24 h. The cell viability was measured using the Cell Counting Kit 8 (WST-8) (Abcam, Cambridge, UK) and incubated with WST-8 solution for 2.5 h at 37 °C and the absorbance was measured at 460 nm according to the manufacturer’s instructions.

### 4.11. Measurement of Lipid Peroxidases

Ferroptosis was induced using RSL3 (2 µM) for 24 h; the cells were then incubated with 200 nM C-11-BODIPY (ThermoFisher Scientific, Waltham, MA, USA) for 30 min at 37 °C and measured using flow cytometry.

### 4.12. Statistical Analyses

Results are presented as mean ± s.d. Statistical analyses of continuous variables were performed using Student’s *t*-test or one-way ANOVA corrected for multiple comparisons. Spearman’s rank correlation analysis was employed to evaluate correlations between the mRNA levels of pairwise genes. The statistical analyses of categorial variables used Fisher’s exact test. Survival analyses were performed using the log-rank (Mantel–Cox) test and corrected for multiple comparisons using the Bonferroni correction. Statistical analyses were performed with Prism 9 software (Graphpad). The survival analysis involved either the time taken for the primary tumor to metastasize (taken from the date of surgery to remove the primary tumor and the date to remove the brain metastasis) or the overall survival once the tumor was in the brain (taken from the date of surgery to remove the brain metastasis and the date of death or last follow-up appointment).

## 5. Conclusions

Δ133p53β was present in the majority of tumors that metastasized to the brain. Δ133p53 was increased in more aggressive tumors and those that more readily metastasized, suggesting Δ133p53 promoted aggressive tumors by upregulating cell surface signaling and entry into the brain, reducing the overall time to metastasis.

## Figures and Tables

**Table 1:** Patient demographics of the brain metastasis cohort.

Brain Metastasis Type	Sex	Age Years (95% CI) *	Primary Tumor Subtype
Breast	Female *n* = 27 (100%)	54.4 (49.5–59.5)	Her2- and ER-positive 10 (37%)
			Her2-positive and ER-negative 4 (14.8%)
	Male *n* = 0 (0%)		Her2-negative and ER-positive 3 (11%), triple-negative 10 (37%)
Colorectal	Female *n* = 9 (43%)	61.8 (53.9–70.7)	Stage T4 1 (11%), stage T3 5 (56%), stage T2 3 (33%), unknown 1 (11%)
	Male *n* = 12 (57%)	61 (53.5–68.5)	Stage T4 6 (50%), stage T3 3 (25%), stage T2 2 (17%), unknown 1 (8%)
Lung	Female *n* = 24 (52%)	63.5 (58.7–68.2)	
	Male *n* = 22 (48%)	61.6 (56.3–66.8)	
Melanoma	Female *n* = 17 (37%)	57.4 (54.5–60.3)	
	Male *n* = 29 (63%)	58.2 (55.5–60.9)	

* Age in years at diagnosis of the brain tumor.

## Data Availability

Raw files for RNA sequences (FASTQ format) were deposited in the Gene Expression Omnibus with accession number GSE221282.

## Supplementary Materials

The supporting information can be downloaded at: https://www.mdpi.com/article/10.3390/ijms24021267/s1.
