# Supplementary material for: Increased Expression of the Δ133p53β Isoform Enhances Brain Metastasis
Source: Int J Mol Sci. 2023 Jan 9;24(2):1267. doi: 10.3390/ijms24021267 (PMC9866425; doi:10.3390/ijms24021267)
Supplement: Supplementary file 1 [file ijms-24-01267-s001.zip › Supplementary Figures.pdf]

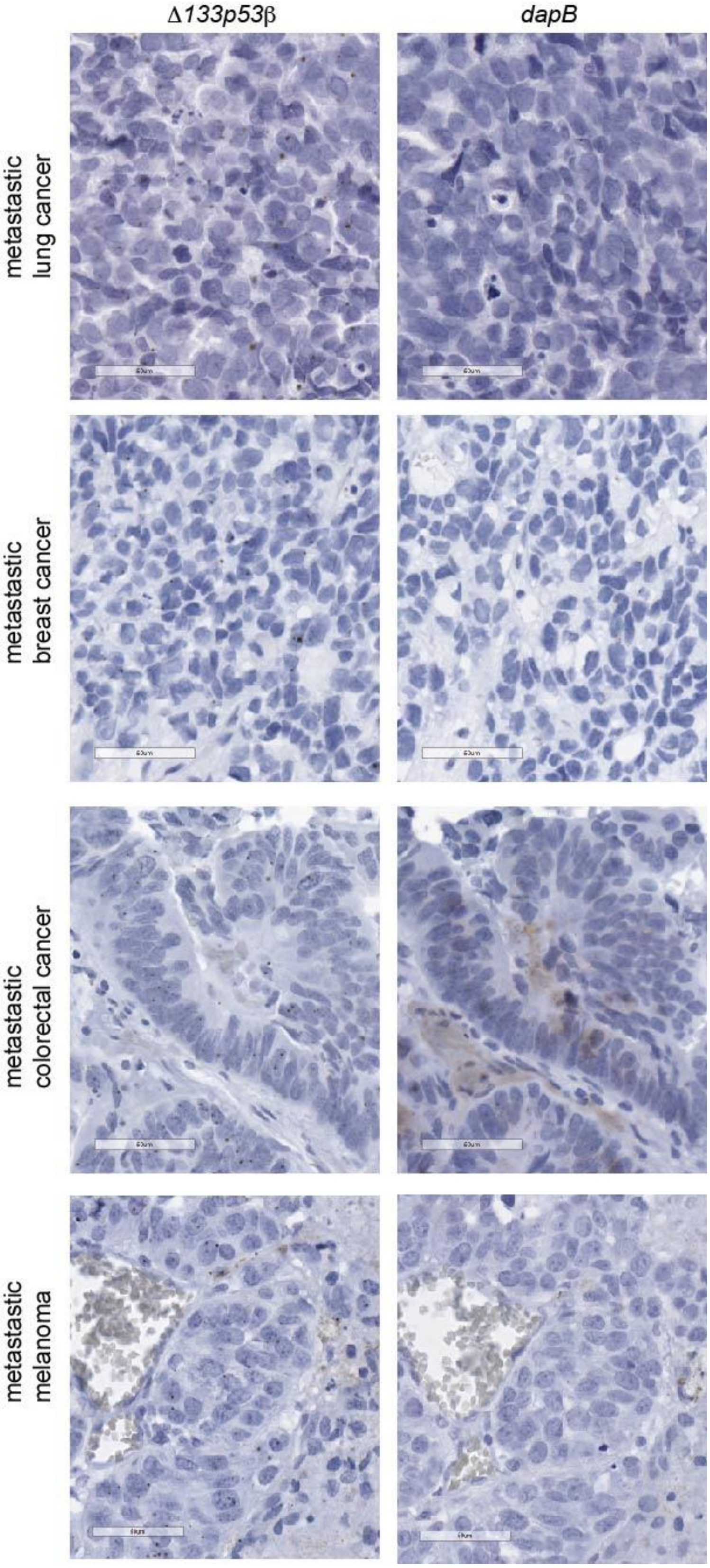

Supplementary Figure S1. Additional examples of *Δ133p53β* mRNA expression in brain metastases. RNAscope was used to detect *Δ133p53β* mRNA and *dapB* was used as a negative control. Images taken at 400x magnification. Scale bar, 50 μm.

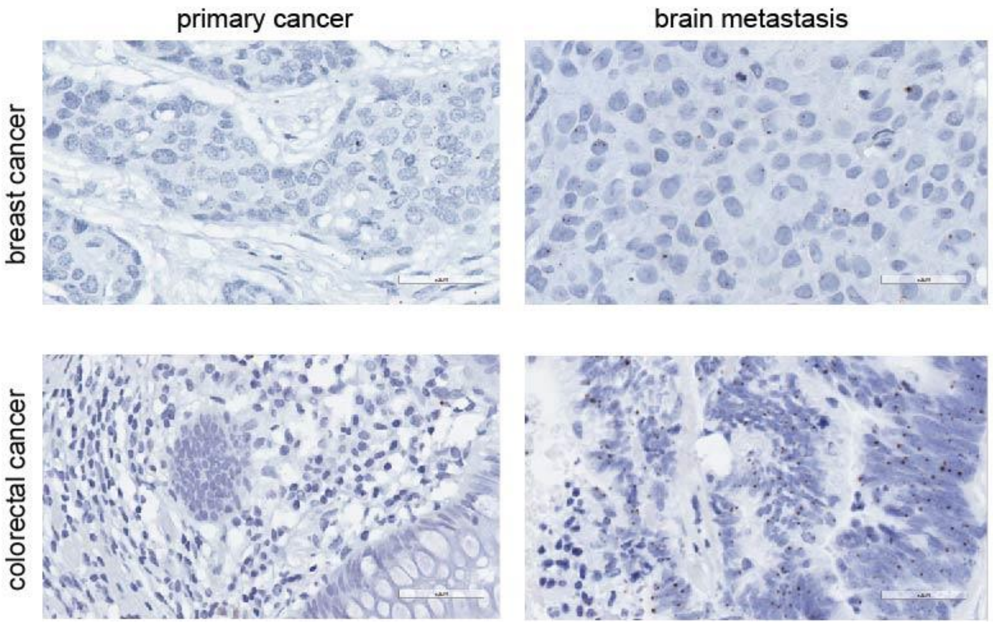

Supplementary Figure S2. Additional examples of *Δ133p53β* mRNA expression in brain metastases and the corresponding primary tumor. Images taken at 400x magnification. Scale bar, 50 μm.
